# Changes in opioid prescribing during the COVID-19 pandemic in England: cohort study of 20 million patients in OpenSAFELY-TPP

**DOI:** 10.1016/S2468-2667(24)00100-2

**Published:** 2024-07-01

**Authors:** Andrea L Schaffer, Colm D Andrews, Andrew D Brown, Richard Croker, William J Hulme, Linda Nab, Jane Quinlan, Victoria Speed, Christopher Wood, Milan Wiedemann, Jon Massey, Peter Inglesby, Seb CJ Bacon, Amir Mehrkar, Chris Bates, Ben Goldacre, The OpenSAFELY Collaborative, Alex J Walker, Brian MacKenna

**Affiliations:** 1 Bennett Institute for Applied Data Science, Nuffield Department of Primary Care Health Sciences, University of Oxford, Oxford, UK; 2 Oxford University Hospitals Trust, Oxford, UK; 3 King’s Thrombosis Centre, King’s College Hospital, London, UK; 4 TPP, TPP House, Leeds, UK

**Keywords:** opioids, covid-19, sars-cov-2, pain, primary care

## Abstract

**Background:**

The COVID-19 pandemic disrupted healthcare delivery, including difficulty accessing in-person care, which may have increased the need for strong pharmacological pain relief.

**Methods:**

With NHS England approval, we used routine clinical data from >20 million general practice adult patients in OpenSAFELY-TPP. Using interrupted time series analysis, we quantified prevalent and new opioid prescribing prior to the COVID-19 pandemic (January 2018-February 2020), and during the restriction (March 2020-March 2021) and recovery periods (April 2021-June 2022), overall and stratified by demographics (age, sex, deprivation, ethnicity, geographic region) and to people in care homes identified via an address-matching algorithm.

**Outcomes:**

There was little change in prevalent prescribing during the pandemic, except for a temporary increase in March 2020. We observed a 9.8% (95%CI -14.5%, -6.5%) reduction in new opioid prescribing from March 2020, with a levelling of the downward trend, and rebounding slightly after April 2021 (4.1%, 95%CI -0.9%, 9.4%). Opioid prescribing rates varied greatly by demographics, but we found a reduction in new prescribing for all subgroups except people 80+ years. Among care home residents, in April 2020 parenteral opioid prescribing increased by 186.3% (95%CI 153.1%, 223.9%).

**Interpretation:**

Opioid prescribing increased temporarily among older people and care home residents, likely reflecting use to treat end-of-life COVID-19 symptoms. Despite vulnerable populations being more impacted by healthcare disruptions, disparities in opioid prescribing by most demographic subgroups did not widen during the pandemic. Further research is needed to understand what is driving the changes in new opioid prescribing and its relation to changes to health care provision during the pandemic.

**Funding:**

The OpenSAFELY Platform is supported by grants from the Wellcome Trust (222097/Z/20/Z) and MRC (MR/V015737/1, MC_PC_20059, MR/W016729/1). In addition, development of OpenSAFELY has been funded by the Longitudinal Health and Wellbeing strand of the National Core Studies programme (MC_PC_20030: MC_PC_20059), the NIHR funded CONVALESCENCE programme (COV-LT-0009), NIHR (NIHR135559, COV-LT2-0073), and the Data and Connectivity National Core Study funded by UK Research and Innovation (MC_PC_20058) and Health Data Research UK (HDRUK2021.000). The views expressed are those of the authors and not necessarily those of the NIHR, NHS England, UK Health Security Agency (UKHSA) or the Department of Health and Social Care.

## Background

In England, 13% of adults received an opioid prescription in 2017/18.^1^ While opioids are effective at treating acute pain, cancer pain, and end-of-life pain, they are commonly overprescribed for chronic non-cancer pain,^2^ where opioids lack evidence of efficacy^3,4^ and are not recommended.^5^ During the COVID-19 pandemic, there were disruptions to provision of healthcare, including access to medicines, primary care appointments, and elective procedures. These disruptions were not experienced equally, with women, people living in deprived areas, and older people most impacted,^6^ the same populations disproportionately affected by opioid-related harms.^7,8^

International studies quantifying opioid prescribing during the COVID-19 pandemic have identified changes not consistent with best practice. A Canadian study found increases in prescribing to people living in care homes,^9^ a population known to be at high risk of opioid-related harms. A US study identified a shift from non-pharmacological treatment (e.g. physical therapy) towards opioid therapy for people with pain,^10^ likely due to increasing remote care during the pandemic. Furthermore, data suggest that rates of opioid-related death and overdose were greater than expected during the pandemic in Canada.^11^

NHS England, the body with national responsibility for care, issued instructions to improve opioid use in 2023, highlighting the need for better use of data to identify, prevent and reduce opioid harm.^12^ While changes in prescribing have been described during COVID-19 in the UK for different classes of medicines^13^, for opioids in specific populations^14,15^ and in aggregate prescribing data^16^, there are no studies on changes to opioid prescribing at the person-level in the general population or in high-risk demographic groups. Due to the risks associated with overprescribing of opioids, especially to vulnerable populations, we aimed to quantify changes to the following measures during the COVID-19 pandemic, overall and by key subgroups: 1) prevalent opioid prescribing; 2) new prescribing; 3) variation in COVID-19-related changes by demographic subgroups and people in care homes.

## Methods

### Study design

We conducted an interrupted time series analysis study (January 2018 to June 2022) using primary care data in England managed by the GP software provider TPP, linked to Office of National Statistics (ONS) death data through OpenSAFELY as part of the NHS England OpenSAFELY COVID-19 service. An overview of OpenSAFELY is available in Nab et al.^17^ We defined two change points: the start of the “restrictions period”, defined as March 2020 as the UK first introduced restrictions on 26 March, and the start of the “recovery period”, defined as April 2021. April 2021 was chosen as it coincides with the start of gradual reopening of non-essential services.^18^

### Participants

We identified all people prescribed an opioid in each month of the study period (January 2018 to June 2022). All people aged ≥18 years, alive, and registered with a TPP practice on the first of every month were included in the denominator for calculation of rates. The TPP population is broadly representative of the full population of England in terms of age, sex, IMD and ethnicity.^19^ We excluded people with missing or impossible values of age and sex (<0.01%) as this is indicative of poor data quality.

### Procedures

Opioids were defined as all medicines falling under the British National Formulary (BNF) legacy paragraphs 4.7.2 (Opioid analgesics), as well as opioid medicines falling under 3.9.1 (Cough suppressants), and opioid-containing combination medicines under 4.7.1 (Non-opioid analgesics),1.4.2 (Antimotility drugs), and 10.1.1 (Non-steroidal antiinflammatory drugs). Opioids used to treat opioid use disorder were not included. Links to the codelists used in this study are openly available for inspection and re-use in this study’s Github repository (https://github.com/opensafely/opioids-covid-research).

Our primary outcome was opioid prescribing prevalence. This was defined as the number of people prescribed an opioid and included both new and repeat prescriptions. The secondary outcome was new opioid prescribing, defined as people prescribed an opioid without any opioid prescription in the previous year. We also identified prescribing of two other opioid subtypes. The first is high-dose long-acting opioids which are not recommended for chronic non-cancer pain.^3^ Among long-acting opioids, high dose opioids were defined as those with ≥120 mg morphine equivalents per day based on the typical total daily dose.^20^ The second is parenteral opioids (i.e. delivered by injection or intravenously), recommended to treat end-of-life symptoms (e.g. pain, breathlessness) in the community.^21^ We hypothesised that an increase in COVID-19 mortality would be associated with an increase in medicines used in palliative care.

First, we characterised people prescribed an opioid between the last three months of the study period (April-June 2022). Opioid prescribing rates were expressed as the number of people prescribed an opioid per 1000 registered adult patients. To prevent disclosure, all counts <=10 have been redacted and rounded to the nearest 7. We included the following demographic categories: sex (male, female); age (18-39, 40-49, 50-59, 60-69, 70-79, 80-89, 90+ years); Index of Multiple Deprivation (IMD) deciles; practice region (East, East Midlands, London, North East, North West, South East, South West, West Midlands, Yorkshire and the Humber); and ethnicity (White [British, Irish, Other]; Asian or Asian British [Bangladeshi, Indian, Pakistani, Other]; Black or Black British [African, Caribbean, Other]; Mixed [White/Asian, White/Black African, White/Black Caribbean, Other]; Other [Chinese, Other]). To compare overall prescribing rates within relevant demographic categories we standardised opioid prescribing rates by age (5-year age bands) and sex using the ONS mid-year 2020 English population.^22^

As there is no flag in the data to identify people residing in care homes (a vulnerable population during the pandemic) we used a combination of coded events (e.g., identification of consultations occurring in care homes) and linking patients’ registered address to care homes as held by the Care Quality Commission, refined by applying the algorithm described by Schultze et al.^23^ Address-based matching has a good positive predictive value.^24^

### Statistical analysis

We estimated changes in monthly opioid prescribing during the restriction and recovery periods using interrupted time series analysis. This approach estimates changes accounting for pre-existing trends. We used the crude (unstandardised) rates for these analyses, as relative changes which would not be affected by standardisation, and due to the additional lack of precision of standardised estimates.

We modelled the number of people prescribed an opioid using negative binomial log-linear regression and included the natural log of the number of registered patients in each month as an offset. The models included variables representing the pre-COVID-19 trend (slope), a level shift (immediate, sustained change) and a slope change (gradual change in trend) after the start of the restriction and recovery periods. The slope change and level shift after the start of the restriction period represent the changes compared with the predicted values had pre-COVID-19 trends continued. The slope change/level shift after the start of the recovery period represent the changes compared with the predicted values had the changes (if any) observed during the restriction period continued. We calculated Newey-West standard errors to account for residual autocorrelation and included dummy month variables to account for seasonality.

Due to reports of increases in opioid sales in March 2020 likely related to stockpiling followed by decreases in April and May,^25^ we tested the model described above for inclusion of dummy variables representing these months to distinguish between temporary and longer-term effects. We retained these dummy variables if they improved model fit determined by the likelihood ratio test. We estimated incidence rate ratios (IRRs) and 95% confidence intervals (CIs) which were expressed as percent changes.

We quantified changes in new prescribing as described above. Here the offset was the number of opioid-naive registered patients in each month (i.e., people without any opioid prescription in the previous year). We also quantified changes in prescribing among people living in care homes. For the outcome of high-dose long-acting prescribing to people in care homes, we used Poisson log-linear regression (instead of negative binomial) as this was a better fitting model based on the likelihood-ratio test.

To estimate differences in prescribing by demographic subgroups, we created separate models for each variable using the same categories stated above for age, sex, IMD decile, ethnicity, and region. People with missing values for IMD and region were excluded from this analysis due to small counts, while people with missing (unknown) ethnicity were treated as a separate group. We tested an interaction term between the level shift and change in slope and each category. As inclusion of an interaction with the change in slope did not improve model fit for any of the subgroups, this interaction term was not retained. We therefore assumed a common trend and that the change in slope did not vary across groups, and only the level shift varied.

As most concerns over opioid prescribing focus on use for chronic non-cancer pain, we repeated our primary (prevalent prescribing) and secondary analyses (new prescribing) excluding people with a cancer diagnosis in the past 5 years as a sensitivity analysis.

### Ethics approval

This study was approved by the Health Research Authority (Research Ethics Committee reference 20/LO/0651) and by the London School of Hygiene and Tropical Medicine Ethics Board (reference 21863).

### Role of the funding source

The funders had no role in the study design; collection, analysis, and interpretation of data; writing of the report; and in the decision to submit the paper for publication.

## Results

From April to June 2022, there were 20,476,680 registered patients (≥18 years) with 1,445,122 prescribed an opioid, or 70.6 per 1000 registered patients. Opioid prescribing increased with age, ranging from 12.6 per 1000 people aged 18-29 years to 202.8 per 1000 people aged 90+ years (Table 1). Prescribing also increased with greater deprivation varying more than two-fold, ranging from 47.7 per 1000 for people in the least deprived IMD decile to 102.6 per 1000 for the most deprived. However, after age and sex standardisation these differences widened further, ranging from 42.0 per 1000 to 120.2 per 1000.

Age and sex standardised rates of opioid prescribing were also high in women (84.9 per 1000), people with Pakistani, Bangladeshi, White British and White Irish ethnicity (95.4, 86.7, 77.0 and 71.8 per 1000), and people living in the North East, North West and West Midlands (89.2, 86.6 and 86.9 per 1000). Differences by ethnicity were attenuated after age and sex standardisation. Among people residing in care homes (0.8% of all registered adult patients), nearly 1 in 4 were prescribed an opioid during this period (228.5 per 1000).

**Figure 1:**
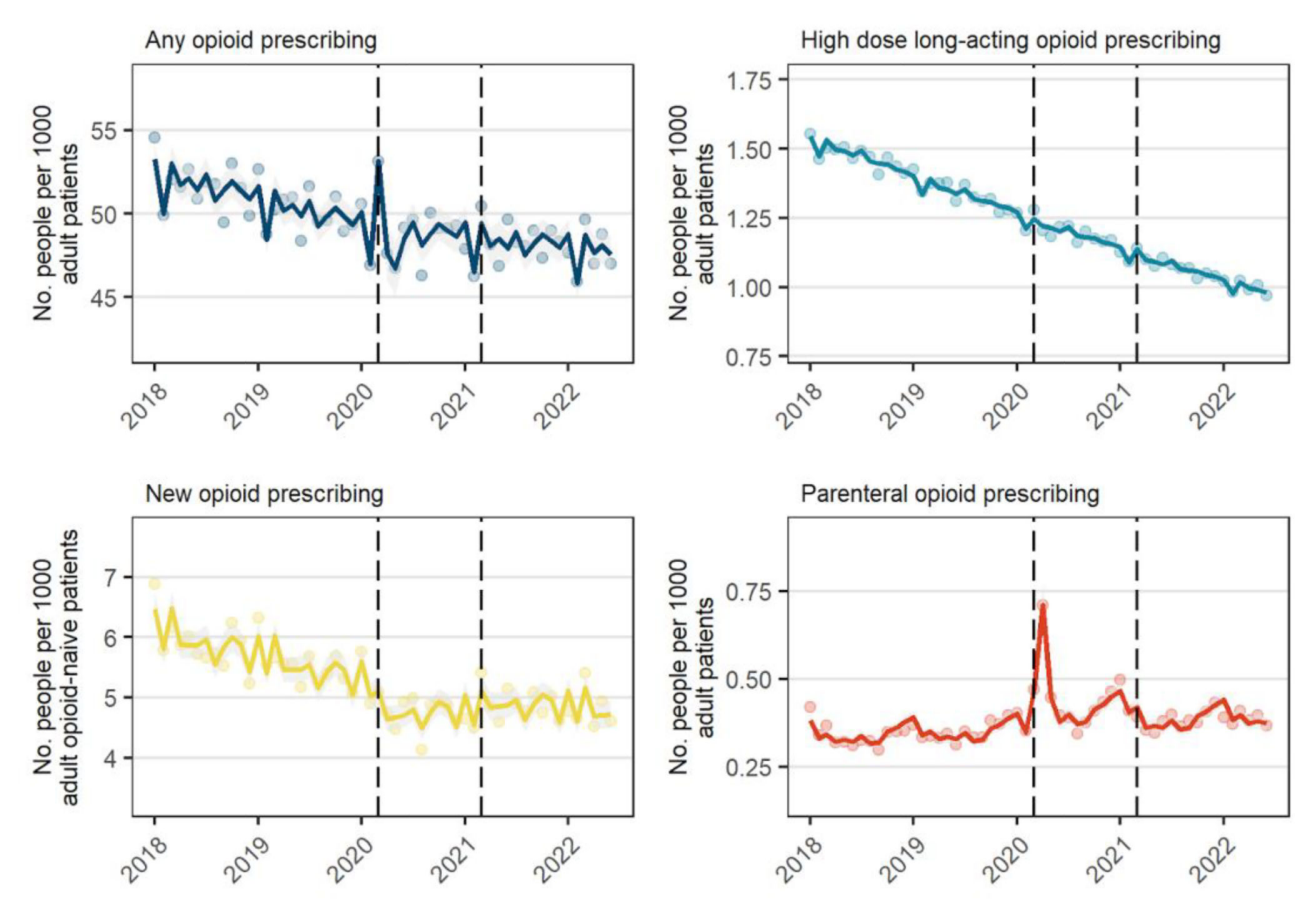
Number of people prescribed opioids per month (Jan 2018 to June 2022) among all registered adult patients. Solid lines are fitted values, dots are observed values, and vertical dashed lines represent start of restriction period (Mar 2020) and recovery period (Apr 2021).

Considering trends over time, there were 19,113,668 registered adult patients in January 2018 increasing over the study period to 20,510,959 in June 2022 (Supplementary Figure 1). The median prevalence of opioid prescribing was 50.9 per 1000 adult patients per month (interquartile range [IQR], 49.6 to 51.7) prior to COVID-19, and was declining by an estimated 0.3% per month (95%CI -0.3%, -0.2%) (Figure 1, Table 2). In March 2020, opioid prescribing prevalence was 7.0% higher than predicted had previous trends continued (95%CI 3.3%, 10.9%); this was followed by lower-than-expected rates in May (-4.7%, 95%CI -7.7%, -1.6%). Aside from these temporary pulses, no changes to the level or slope were observed during the restriction or recovery periods. Similar results were observed when excluding people with a cancer diagnosis in the past 5 years (Supplementary Figure 2, Supplementary Table 1).

In each month, a median of 9% of all opioid prescriptions were new prescriptions. There was a median of 5.7 people newly prescribed opioids per 1000 opioid-naïve patients per month (IQR, 5.4 to 5.9) and was declining by 0.6% per month pre-COVID-19 (95%CI -0.7%, -0.5%) (Figure 1, Table 2). In contrast to prevalent prescribing, no increase was observed in March 2020. Starting during the restriction period, there was a -9.8% level shift in new prescribing (95%CI -14.5%, -6.5%) and a 0.6% increase in slope (95%CI 0.2%, 1.1%) and a small, non-significant, upward shift during the recovery period relative to the restriction period (4.1%, 95%CI -0.9%, 9.4%).

High-dose long-acting opioids represented a small minority of opioid prescribing. The median prescribing prevalence was 1.4 per 1000 per month pre-COVID-19 (IQR, 1.4 to 1.5) and was declining by 0.8% per month (95%CI -0.9%, -0.8%). No changes were observed during the restriction or recovery periods. For parenteral opioids, the median prevalence was 0.4 per 1000 per month pre-COVID-19 (IQR, 0.3 to 0.4). However, there were large increases in prescribing in March-May 2020, including a 18.0% (95%CI 6.1%, 31.2%) increase in March, a 89.4% (95%CI 76.0%, 103.8%) increase in April, and a 16.8% (95%CI 8.3%, 26.0%) increase in May. Even after accounting for these temporary increases, a positive level shift was observed during the restriction period (10.7%, 95%CI -0.4%, 23.1%), which reduced during the recovery period relative to the restriction period (-8.4%, 95%CI -14.6%, -1.8%).

**Figure 2:**
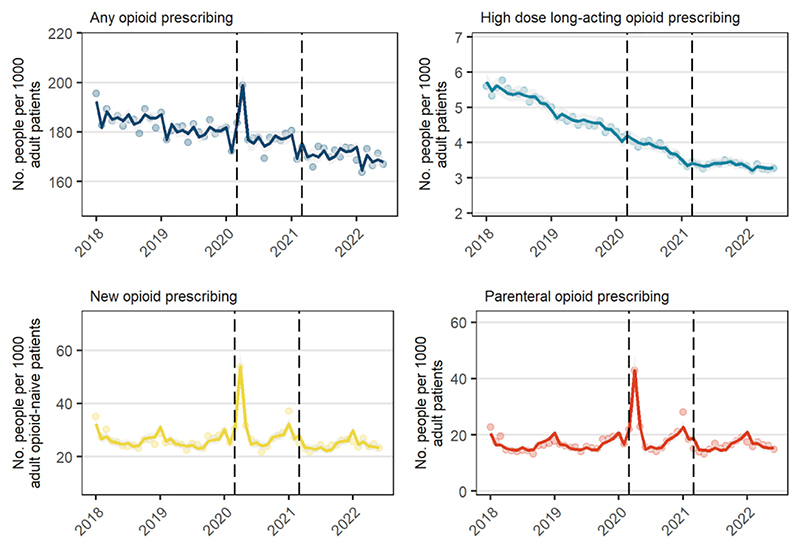
Number of people prescribed opioids per month (Jan 2018 to Mar 2022) among registered patients living in care homes. Solid lines are fitted values, dots are observed values, and vertical dashed lines represent start of restriction period (Mar 2020) and recovery period (Apr 2021).

There was a median of 155,943 registered adult patients living in a care home per month (IQR, 151,298 to 158,774). Prior to the start of COVID-19 period, a median of 182.4 people were prescribed an opioid per 1000 patients in a care home (IQR, 180.2 to 185.1), which declined by 0.2% per month (95%CI -0.3%, -0.2%) (Figure 2, Table 2). An increase in prevalent prescribing was observed in March (2.9%, 95%CI 0.3%, 5.6%) and April (13.3%, 95%CI 11.2%, 15.4%), and there was a small negative level shift during the recovery period (-1.5%, 95%CI -3.0%, -0.01%). Prescribing of high dose, long-acting opioids also declined by 1.3% per month (95%CI -1.4%, -1.1%), with an increasing slope starting in the recovery period (1.5%, 95%CI 1.3%, 1.8%).

Median new opioid prescribing was 25.4 per 1000 opioid-naïve patients in a care home pre-COVID-19 (IQR, 24.5 to 27.3) and was stable. Increases in new prescribing were observed in April (112.5%, 95%CI 92.2%, 134.9%) and May (26.0%, 95%CI 14.6%, 38.5%). After accounting for these changes, no other changes were observed during the restriction period. There was a -10.2% level shift (95%CI -18.7%, -0.7%) in new prescribing starting in the recovery period. Prescribing of parenteral opioids was much higher in care homes than in the general population (16.2 per 1000 pre-COVID-19) and there were large increases in prescribing in March-May 2020, including a 35.9% (95%CI 11.6%, 65.5%) increase in March, 186.3% (95%CI 153.1%, 223.9%) in April, and 54.2% (95%CI 37.0%, 73.7%) in May. Aside from these temporary changes, there was also a level shift during the recovery period relative to the restriction period of -14.3% (95%CI -26.7%, 0.2%).

**Figure 3:**
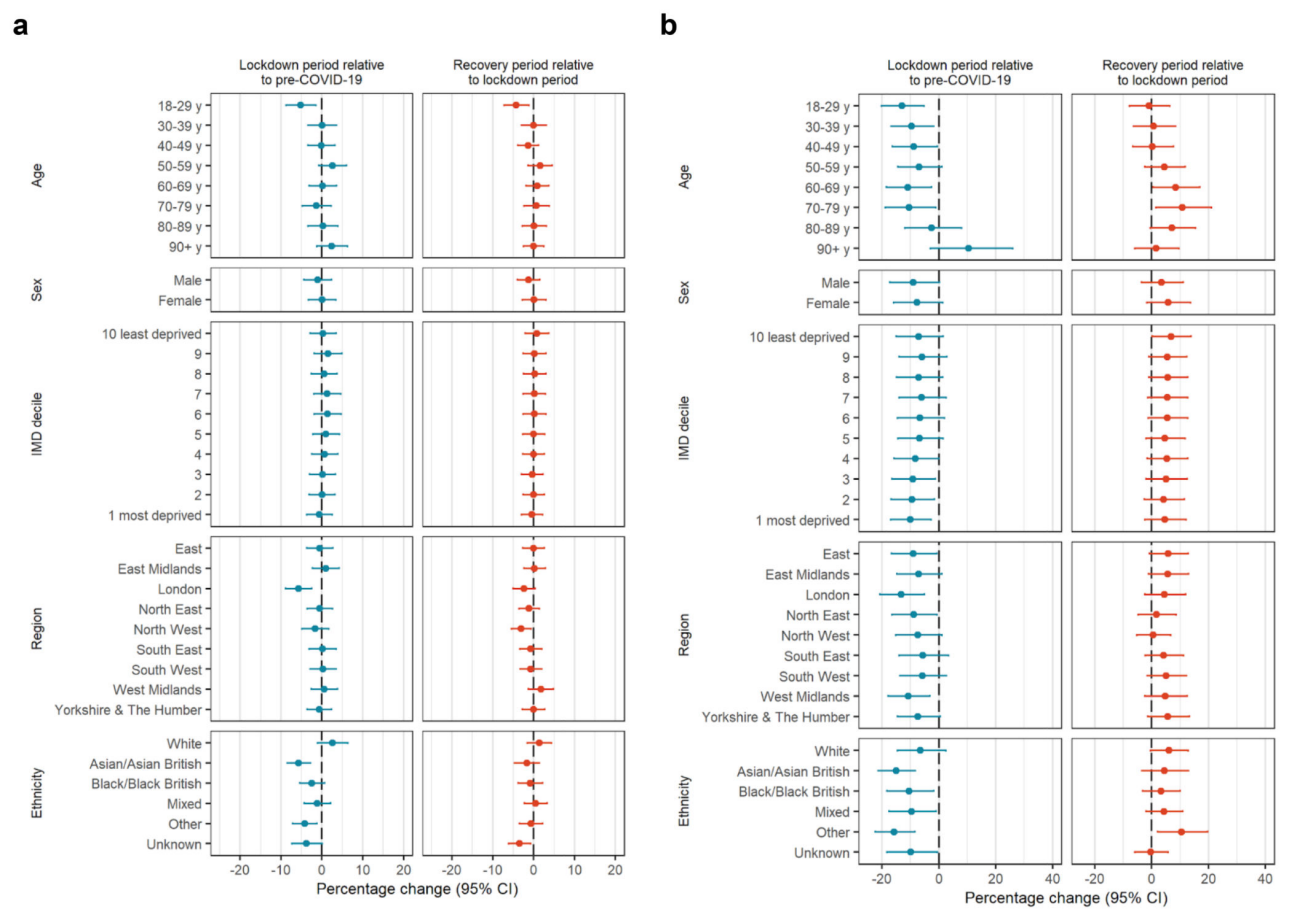
Changes in number of people prescribed opioids (**a**) and newly prescribed opioids (**b**) during the restriction and recovery periods by demographics, adjusted for long-term trends and seasonality

Demographic variation in prevalent and new opioid prescribing by month (Supplementary Figure 3, Supplementary Figure 4) mirrored those observed in Table 1. During the restriction period, there was a negative shift in overall opioid prescribing for people aged 18-29 years (-5.2%, 95%CI -8.9%, -1.4%) compared with predicted values had previous trends continued, but no change for all other age groups (Figure 3a, Supplementary Table 2). Other differences include a decrease in people with Asian or Asian British ethnicity (-5.7%, 95%CI -8.5%, -2.8%), Other ethnicity (-4.2%, 95%CI -7.2%, -1.2%) and people living in London (-5.8%, 95%CI -8.9%, -2.5%). These decreases did not reverse during the recovery period. For people aged 18-29 years, there was a further level shift of -4.2% (95%CI -7.2%, -1.2%) during the recovery period compared with the restriction period. There was little variation by sex or IMD decile.

For new opioid prescribing, no significant differences were observed by sex. The change in new prescribing associated with the restriction period varied most dramatically by age (Figure 3b, Supplementary Table 3). There were negative shifts in new prescribing ranging from -7.0% to -13.0% in age categories <80 years; these decreases reversed in the recovery period compared with the restriction period for people aged 60-79 years, but not in younger age groups. We observed an increase in new prescribing in April 2020 for people 90+ years compared with predicted values had previous trends continued, which was not observed for other age groups. For most other demographic categories, there were similar decreases in new prescribing during the restriction period, with minimal evidence of rebounding during the recovery period.

## Discussion

While the COVID-19 pandemic was associated with minimal changes in prevalent opioid prescribing in England, we found decreases in people newly prescribed opioids, with a levelling of the downward trend and some evidence that the reduction reversed slightly during the recovery period. And while our findings confirm previously identified differences in opioid prescribing by deprivation, ethnicity, and geography,^7,20^ we found only minimal differences in how the pandemic impacted on opioid prescribing by sex, IMD decile, region, and ethnicity. The exception was older people and people living in care homes where temporary increases in prescribing of parenteral opioids coincided with the peak in COVID-19 morbidity and mortality, strongly suggesting use to treat end-of-life COVID-19 symptoms.^21^

Our work also confirms continuation of the downward trend in opioid prescribing in England starting in 2016 seen in aggregate prescribing data^20^, coinciding with a policy focus on reducing opioid prescribing for chronic non-cancer pain.^26^ One other study also found a decrease in new opioid users among people with certain musculoskeletal or rheumatic conditions, but not in the number of overall prescriptions.^14^ This reduction was attributed by those authors to caution from GPs in newly prescribing opioids during the pandemic when monitoring was more difficult. Early in the pandemic, there was also a decrease in elective medical procedures that often require prescribed analgesia^27^; however, opioids would typically be supplied by the hospital and are not captured in our data. Therefore, reduced new opioid prescribing is more likely related to decreased primary care contacts and fewer opportunities for opioids to be initiated.^28^

We identified higher prescribing rates among women compared with men, although both groups were impacted similarly during the pandemic. Higher rates of analgesic use among women are consistently found in UK studies,^29,30^ although rates of long-term use tend to be more similar between the sexes.^29^ The cause is likely multifactorial, including differences in chronic pain prevalence, pain tolerance, sensitivity to analgesia, and propensity to seek or receive treatment.^31–33^ Similarly, prescribing varied substantially by ethnicity, region and IMD. People living in areas of greater deprivation have higher rates of suboptimal opioid prescribing such as long-term use^7,8^ in part related to variation in rates of chronic pain.^34^ While people living in deprived areas experienced greater healthcare disruption and worse outcomes during the pandemic,^6,35^ the disparity in opioid prescribing did not widen further.

We could not definitely identify people who were at end-of-life, however parenteral opioids are recommended in palliative care including for treating severe COVID-19 and the spikes in parenteral opioid prescribing both in the general population and people in care homes coincide with the peak in mortality during the pandemic (Supplementary Figure 5)^36^ strongly suggesting this is related to treatment of people at the end-of-life..^37^ Most of the peak in parenteral opioid prescribing was attributable to people in care homes who were greatly impacted during the pandemic; in the first 12 weeks one third of all deaths in care homes were attributable to COVID-19.^38^ A similar pattern was observed with antipsychotic prescribing.^39^ While opioid prescribing for treatment of patients at the end-of-life is best practice^40^ other studies have identified increases in prescribing of sedating medicines (opioids, antidepressants, antipsychotics, benzodiazepines) to people in care homes,^9^ to deal with the psychological symptoms resulting from increased social isolation during the pandemic.^41^

Our data capture primary care records for approximately 40% of patients registered with a practice in England via the OpenSAFELY platform, and these data are broadly representative of the wider English population.^19^ This study is the largest to quantify person-level changes in opioid prescribing during the COVID-19 pandemic in England, allowing us to identify changes by demographic groups and in new prescribing, which is not possible with aggregate data. While understanding how opioid prescribing has changed is important, it is limited in what it tells us about the quality of prescribing. We don’t know the reason for prescribing, as is common with many large database studies. We did not have information on prescribed dose or duration which could identify more nuanced changes such as dose escalation as this is not currently captured in a structured format on prescriptions in England. While we included all opioids (including non-analgesia), most prescribing in England is for weak opioids for pain (e.g. codeine). Weak opioids are generally less harmful than stronger opioids, but there are still risks if used inappropriately.^42^

There is no gold standard to identify people living in care homes in England, and we have relied on a previously developed algorithm that uses a combination of coded procedures and address-matching.^23^ Algorithms based on address have good positive predictive value for identifying care home residents,^43^ and the prevalence in our study is similar to ONS data.^44^ Our data also only includes prescriptions in primary care not in secondary care, and some people may have been admitted to hospital where opioid prescribing would not be captured. However, nearly one in four deaths in people aged >70 years during the pandemic was in a care home.^37^ While there will be some underascertainment of opioid prescribing in care homes residents, the relative changes observed are likely to still hold.

Even though we saw no change in prevalent prescribing during the pandemic, there were multiple pressures that may have resulted both in decreased (e.g. fewer interactions with the healthcare system) and increased prescribing (e.g. less availability of non-pharmacological pain treatment). Decreases in new prescribing (which represents a minority of all prescribing) may have been offset by increases in other subgroups. For instance, the COVID-19 pandemic led to worsening of the backlog in elective procedures with nearly 7.0 million people on waiting lists as of August 2022^45^ putting people at increased risk of long-term opioid use and quantifying this impact is vital. Further, while we found no evidence that disparities in prescribing by demographic factors widened during the pandemic, identifying the drivers of these observed differences can help inform strategies to reduce suboptimal use in high-risk populations.

A better understanding of the changes observed in this study is needed. A 2019 report by Public Health England raised concerns about the high prevalence of opioid prescribing, and emphasised the need for quality and contemporary and detailed data on predictors of long term use and dependence^1^ and NHS England highlighted the need for better use of data to reduce opioid harm.^12^ We are developing tools to facilitate near real-time audit and feedback in the context of rapidly evolving pressures on the health service readily extendable to other clinical and challenges and can include any measures on opioids needed to support NHS England’s ambition on safe opioid use.

In conclusion, we found little change in overall opioid prescribing apart from temporary changes at the start of the first lockdown, with changes in new opioid prescribing which rebounded slightly during the recovery period. Disparities in opioid prescribing by demographic factors did not widen during the pandemic. However, we observed a substantial temporary increase in parenteral opioid prescribing and new opioid prescribing for people living at addresses linked to care homes, coinciding with the peak in COVID-19 morbidity and mortality and likely representing use to treat end-of-life COVID-19 symptoms.

## Supplementary Material

Appendix

## Figures and Tables

**Table 1:** Registered adult patients (≥18 years) prescribed an opioid between April and June 2022. All counts rounded to nearest 7.

	No. registered patients	No. prescribed opioids	Prevalence per 1000 population	
	N (% of total)	N (% of patients)	Crude	Age and sex standardised_*_
**Total**	20,476,680 (100.0)	1,445,122 (7.1)	70.6	–
**Sex**				
Female	10,278,870 (50.2)	898,114 (8.7)	87.4	84.9
Male	10,197,810 (49.8)	547,001 (5.4)	53.6	55.2
**Age**				
18-29 y	3,717,679 (18.2)	46,886 (1.3)	12.6	12.8
30-39 y	3,659,908 (17.9)	108,255 (3.0)	29.6	30.0
40-49 y	3,276,966 (16.0)	165,214 (5.0)	50.4	51.2
50-59 y	3,492,216 (17.1)	276,024 (7.9)	79.0	79.7
60-69 y	2,802,520 (13.7)	308,245 (11.0)	110.0	110.2
70-79 y	2,248,085 (11.0)	307,349 (13.7)	136.7	136.1
80-89 y	1,047,270 (5.1)	186,088 (17.8)	177.7	173.8
90+ y	232,036 (1.1)	47,061 (20.3)	202.8	190.1
**IMD decile**				
1 most deprived	1,928,423 (9.4)	197,862 (10.3)	102.6	120.2
2	1,911,868 (9.3)	172,599 (9.0)	90.3	102.1
3	1,932,042 (9.4)	156,940 (8.1)	81.2	88.8
4	2,026,626 (9.9)	149,506 (7.4)	73.8	77.1
5	2,099,167 (10.3)	148,050 (7.1)	70.5	69.8
6	2,223,221 (10.9)	146,265 (6.6)	65.8	62.9
7	2,009,497 (9.8)	125,923 (6.3)	62.7	58.3
8	2,056,257 (10.0)	121,835 (5.9)	59.3	54.7
9	1,993,831 (9.7)	112,651 (5.6)	56.5	50.5
10 least deprived	1,744,274 (8.5)	83,202 (4.8)	47.7	42.0
*Missing*	*551,460 (2.7)*	*30,289 (5.5)*	*54.9*	*67.0*
**Region**				
East	4,652,396 (22.7)	298,844 (6.4)	64.2	64.0
East Midlands	3,552,633 (17.3)	273,147 (7.7)	76.9	76.4
London	1,493,926 (7.3)	47,593 (3.2)	31.9	46.1
North East	938,903 (4.6)	83,090 (8.8)	88.5	89.2
North West	1,766,548 (8.6)	160,160 (9.1)	90.7	86.6
South East	1,360,093 (6.6)	84,441 (6.2)	62.1	59.4
South West	2,926,749 (14.3)	198,870 (6.8)	67.9	63.0
West Midlands	804,657 (3.9)	65,226 (8.1)	81.1	86.9
kshire and The Humber	2,911,692 (14.2)	229,453 (7.9)	78.8	79.6
*Missing*	*69,083 (0.3)*	*4291 (6.2)*	*62.1*	*71.1*
**Ethnicity**				
White	13,732,257 (67.1)	1,087,471 (7.9)	79.2	73.7
British	11,702,222 (57.1)	1,00,3373 (8.6)	85.7	77.0
Irish	99,127 (0.5)	8211 (8.3)	82.8	71.8
Other	1,930,908 (9.4)	75,887 (3.9)	39.3	56.2
Asian or Asian British	1,371,685 (6.7)	58,639 (4.3)	42.7	64.3
Bangladeshi	90,923 (0.4)	4431 (4.9)	48.7	86.7
Indian	580,874 (2.8)	20,188 (3.5)	34.8	51.2
Pakistani	374,626 (1.8)	23,282 (6.2)	62.1	95.4
Other	325,269 (1.6)	10,738 (3.3)	33.0	51.4
Black or Black British	460,236 (2.2)	20,440 (4.4)	44.4	62.9
African	287,581 (1.4)	10,493 (3.6)	36.5	63.7
Caribbean	97,790 (0.5)	6447 (6.6)	65.9	63.4
Other	74,865 (0.4)	3500 (4.7)	46.8	65.0
Mixed	244,097 (1.2)	9401 (3.9)	38.5	62.0
White/Asian	50,351 (0.2)	1687 (3.4)	33.5	57.2
White/Black African	48,160 (0.2)	1764 (3.7)	36.6	61.2
White/Black Caribbean	54,397 (0.3)	2807 (5.2)	51.6	73.3
Other	91,182 (0.4)	3143 (3.4)	34.5	56.6
Other	411,992 (2.0)	11,340 (2.8)	27.5	48.1
Chinese	160,958 (0.8)	1008 (0.6)	6.3	17.6
Other	251,034 (1.2)	10,332 (4.1)	41.2	64.6
*Missing*	*4,256,413 (20.8)*	*257,838 (6.1)*	*60.6*	*66.5*
				
**Living in care home**	168,483 (0.8)	38,493 (22.8)	228.5	118.7

IMD = Index of Multiple Deprivation;

* Age (5-year age bands) and sex-standardised using Office of National Statistics English mid-year 2020 population

**Table 2:** Relative changes in number of people prescribed opioids per 1000 population during the restriction (Mar 2020-Mar 2021) and recovery (Apr 2021-Jun 2022) periods among all registered adult patients and people living in care homes.

	Pre-COVID-19 monthly slope (%, 95% CI)	Changes during restriction period relative to pre-COVID-19					Changes during recovery period relative to restriction period	
		Level shift (%, 95% CI)	Change in slope (%, 95% CI)	March 2020 (%, 95% CI)	April 2020 (%, 95% CI)	May 2020 (%, 95% CI)	Level shift (%, 95% CI)	Change in slope (%, 95% CI)
**Full adult population**								
Any opioid	-0.3 (-0.3, -0.2)	-0.6 (-3.4, 2.5)	0.2 (-0.1, 0.6)	7.0 (3.3, 10.9)	-2.0 (-4.2, 0.3)	-4.7 (-7.7, -1.6)	-0.6 (-3.0, 1.9)	-0.03 (-0.4, 0.3)
New opioid	-0.6 (-0.7, -0.5)	-9.8 (-14.5, -6.5)	0.6 (0.2, 1.1)	*	*	*	4.1 (-0.9, 9.4)	-0.3 (-0.8, 0.2)
High dose long-acting opioid	-0.8 (-0.9, -0.8)	-1.1 (-2.6, 0.5)	0.03 (-0.1, 0.2)	*	*	*	-1.2 (-2.5, 0.1)	-0.03 (-0.2, 0.1)
Parenteral opioid	0.2 (-0.1, 0.5)	10.7 (-0.4, 23.1)	0.2 (-0.8, 1.3)	18.0 (6.1, 31.2)	89.4 (76.0, 103.8)	16.8 (8.3, 26.0)	-8.4 (-14.6, -1.8)	-0.2 (-1.5, 1.1)
								
**People living in care homes**								
Any opioid	-0.2 (-0.3, -0.2)	0.2 (-2.1, 2.4)	0.09 (-0.2, 0.3)	2.9 (0.3, 5.6)	13.3 (11.2, 15.4)	0.3 (-2.3, 2.9)	-1.5 (-3.0, -0.01)	0.05 (-0.2, 0.3)
New opioid	-0.3 (-0.6, 0.04)	4.4 (-9.4, 20.4)	0.5 (-1.0, 2.1)	12.9 (-2.3, 30.6)	112.5 (92.2, 134.9)	26.0 (14.6, 38.5)	-10.2 (-18.7, -0.7)	-0.06 (-1.8, 1.7)
High dose long-acting opioid	-1.3 (-1.4, -1.1)	1.7 (-1.0, 4.5)	-0.4 (-0.7, -0.2)	*	*	*	-0.6 (-2.9, 1.7)	1.5 (1.3, 1.8)
Parenteral opioid	0.03 (-0.5, 0.5)	-4.1 (-21.1, 16.6)	1.2 (-1.1, 3.6)	35.9 (11.6, 65.5)	186.3 (153.1, 223.9)	54.2 (37.0, 73.7)	-14.3 (-26.7, 0.2)	-0.8 (-3.5, 2.0)

* Not included in model

## Data Availability

Primary care records managed by the GP software provider TPP were linked to Office of National Statistics (ONS) death data through OpenSAFELY and were linked, stored and analysed securely within the OpenSAFELY platform: https://opensafely.org/ as part of the NHS England OpenSAFELY COVID-19 service. Data include pseudonymised data such as coded diagnoses, medications and physiological parameters. No free text data are included. Detailed pseudonymised patient-level data is potentially re-identifiable and therefore not shared. The process for external users to request access to the data are available on the OpenSAFELY website (https://www.opensafely.org/). The public Github repository for this project (https://github.com/opensafely/opioids-covid-research) includes: all code used in this study, shared openly for review and re-use under MIT open license; all codelists used in this study; and the study protocol. Detailed information on how each codelist was compiled is available at https://codelists.opensafely.org/ for inspection and re-use by the wider research community.

## Evidence before this study

We searched Pubmed for publications between 1 March 2020 and 8 January 2023 using the following search terms: (“COVID-19” OR “SARS-CoV-2”) AND (“United Kingdom” OR “England” OR “Britain” OR “Scotland” OR “Wales”) AND (“opioid”). We also searched the reference list of relevant articles. We included research studies (excluding conference abstracts and editorials) that quantified opioid prescribing or use in the United Kingdom during the COVID-19 pandemic. Studies focussed solely on opioid substitution therapy for treatment of opioid use disorder were excluded. We considered that studies using simple before-and-after comparisons provided lower quality evidence than study designs that took into account long-term trends such as interrupted time series analysis.

We identified four studies. One performed a before-and-after comparison of opioid use among people on a waiting list for hip or knee arthroplasty in Scotland (n=548) during the COVID-19 pandemic and historical controls and found higher rates of long-term opioid use among people on waiting lists during the pandemic. The second study used a segmented linear regression to quantify changes in opioid prescribing using English Prescription Cost Analysis aggregate prescription data. This study found no changes in opioid prescribing after the start of the COVID-19 pandemic. However, due to the lack of person-level data this study was not able to identify changes in incident use. The third study of 1.3 million people with rheumatic and musculoskeletal diseases used regression analysis to quantify changes over time and found a decrease in new opioid users among people with certain conditions, but not in the number of overall prescriptions. The last study of 34,711 people newly diagnosed with cancer and 30,256 who died of cancer in Wales found increases in strong opioid prescribing in both populations.

## Added value of this study

This is the largest study (>20 million patients) of opioid prescribing during the COVID-19 pandemic in a representative sample of the population of England. We used person-level data to quantify changes in the number of people prescribed opioids and identified that in the general population, prevalent opioid prescribing changed little, with the exception of a temporary increase at the start of the first lockdown. However, we also identified reductions in new opioid prescribing during the restriction period accompanied by a levelling of the downward trend. While our findings confirm previous studies quantifying variation in opioid prescribing by sex, ethnicity, region and deprivation, we showed that changes to new prescribing during the COVID-19 pandemic were experienced approximately similarly across these subgroups. The exceptions were older people and people in care homes, who were identified using a previously developed address-matching algorithm. The latter group experienced substantial temporary increases in opioid prescribing primarily due to parenteral opioids, which are used in palliative care. This coincided with periods of greatest COVID-19 morbidity and mortality in this population.

## Implications of all the available evidence

The COVID-19 pandemic resulted in substantial disruptions to the healthcare system. Despite concerns that difficulty or delays in providing care during the pandemic may have led to shifts from non-pharmacological treatments to greater opioid prescribing, we observed no increases in prescribing prevalence in most demographic groups in England. The one exception is people living at addresses that matched to care homes residences, where the observed prescribing patterns suggest use to treat end of life symptoms, consistent with best practice. However, different populations may have been impacted differently during the pandemic and these effects may have averaged out. While we found no evidence that disparities in prescribing by demographic factors widened during the pandemic, a better understanding of the drivers of this variation is needed. Our findings also do not preclude changes in prescribing in high-risk populations, such as increased prescribing to people on procedure waiting lists.
