# Supplementary material for: Changes in opioid prescribing during the COVID-19 pandemic in England: cohort study of 20 million patients in OpenSAFELY-TPP
Source: Lancet Public Health. Author manuscript; Available in PMC 2024 Sep 30. (PMC7616651; doi:10.1016/S2468-2667(24)00100-2)
Supplement: Appendix [file EMS198173-supplement-Appendix.pdf]

Appendix

The OpenSAFELY Collaborative

Bennett Institute for Applied Data Science

Sebastian CJ Bacon

Lucy Bridges

Benjamin FC Butler-Cole

Simon Davy

Iain Dillingham

David Evans

Louis Fisher

Amelia Green

Ben Goldacre

Liam Hart

George Hickman

Peter Inglesby

Steven Maude

Amir Mehrkar

Thomas O'Dwyer

Rebecca M Smith

Pete Stokes

Tom Ward

Jon Massey

Milan Wiedemann

TPP

Christopher Bates

Jonathan Cockburn

Sam Harper

Frank Hester

John Parry

Supplementary Figures and Tables

Supplementary Figure 1. Number of registered adult patients by month during the study period. Opioid-naive is defined as people with an opioid prescription in the past year. Vertical dash lines represent the start of the lockdown (Mar 1, 2020) and recovery (Apr 1, 2021) periods.

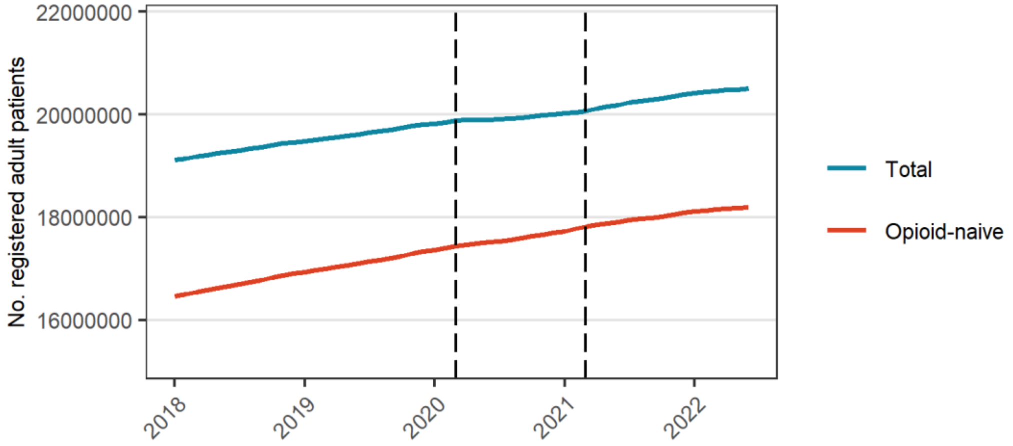

Supplementary Figure 2. Number of people prescribed opioids per month (Jan 1, 2018 to June 30, 2022) among all registered adult patients without a history of cancer. Solid lines are fitted values, dots are observed values, and vertical dashed lines represent start of lockdown period (Mar 1, 2020) and recovery period (Apr 1, 2021).

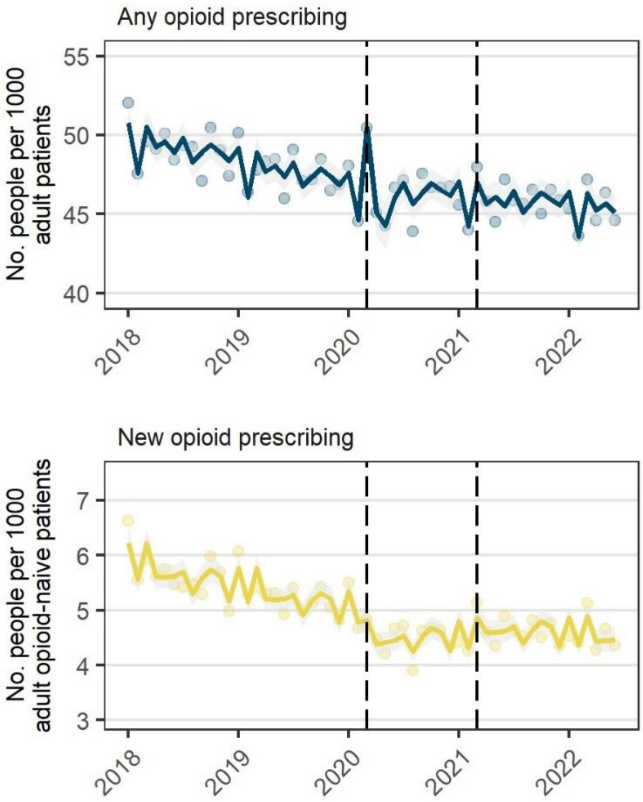

Supplementary Table 1. Relative changes in number of people prescribed opioids per 1000 population during the lockdown (Mar 2020-Mar 2021) and recovery (Apr 2021-Jun 2022) periods among all registered adult patients without a history of cancer.

	**Pre-COVID-19 monthly slope (%, 95% CI)**	**Changes during lockdown period relative to pre-COVID-19**					**Changes during recovery period relative to lockdown period**	
		**Level shift (%, 95% CI)**	**Change in slope (%, 95% CI)**	**March 2020 (%, 95% CI)**	**April 2020 (%, 95% CI)**	**May 2020 (%, 95% CI)**	**Level shift (%, 95% CI)**	**Change in slope (%, 95% CI)**
**Full adult population**								
Any opioid	-0.3 (-0.3, -0.2)	-0.6 (-3.5, 2.3)	0.2 (-0.1, 0.6)	7.0 (3.3, 10.9)	-2.2 (-4.4, 0.07)	-4.9 (-7.9, -1.8)	-0.6 (-3.5, 2.3)	-0.1 (-0.4, 0.3)
New opioid	-0.6 (-0.8, -0.5)	-10.2 (-13.3, -7.1)	0.7 (0.3, 1.1)	*	*	*	4.1 (-0.8, 9.3)	-0.4 (-0.8, 0.1)

*Not included in model

Supplementary Figure 3. Number of people prescribed opioids per month (Jan 1, 2018 to Jun 30, 2022) among all registered adult patients, by: a. age category; b. sex; c. IMD decile; d. ethnicity; e. region. Solid lines are fitted values, dots are observed values, and vertical black lines represent the start of lockdown period (Mar 1, 2020) and recovery period (Apr 2, 2021).

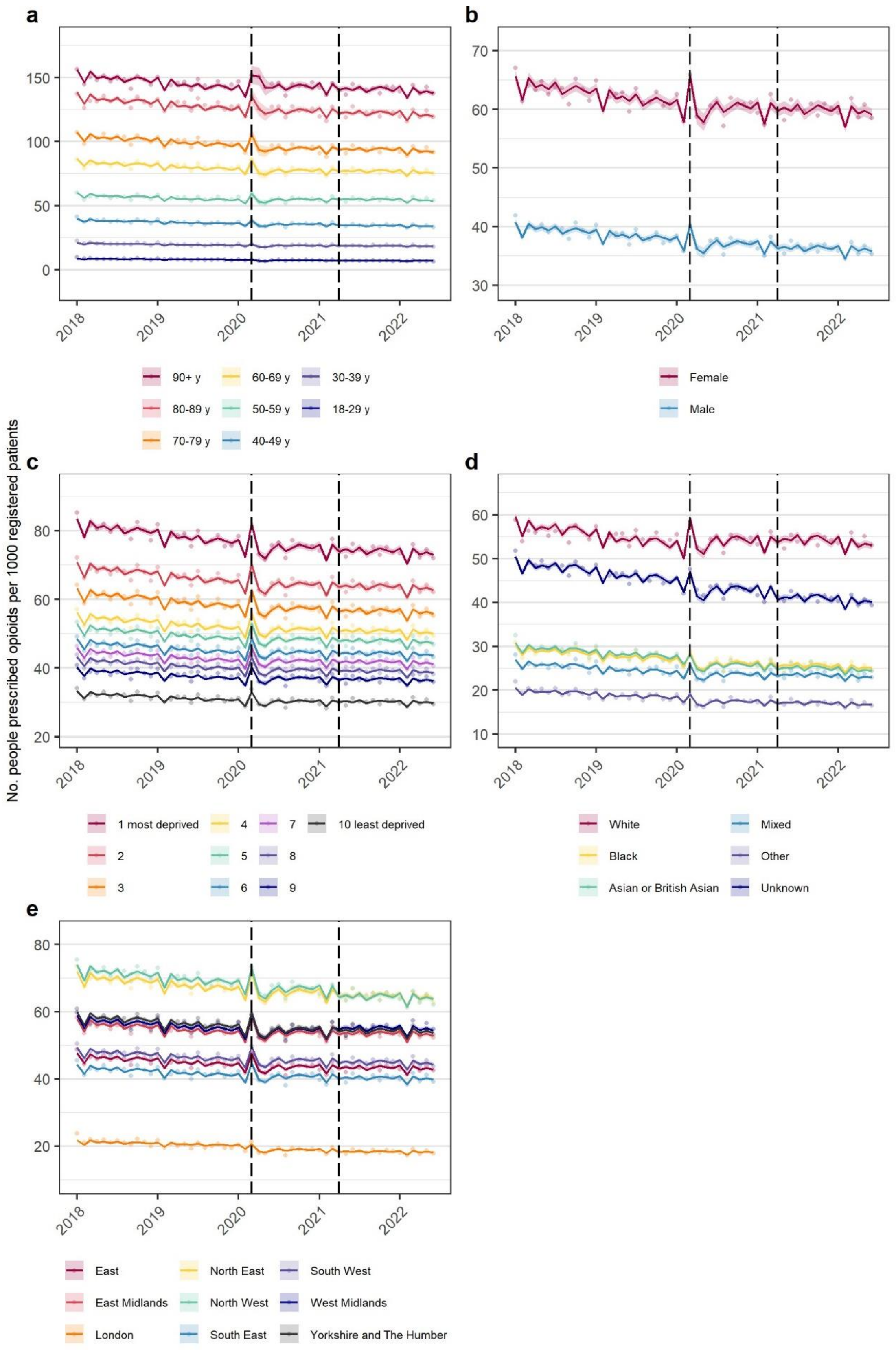

Supplementary Figure 4. Number of people newly prescribed opioids per month (Jan 1, 2018 to Jun 30, 2022), among all registered adult patients, by: a. age category; b. sex; c. IMD decile; d. ethnicity; e. region. Solid lines are fitted values, dots are observed values, and vertical black lines represent the start of lockdown period (Mar 1, 2020) and recovery period (Apr 1, 2021).

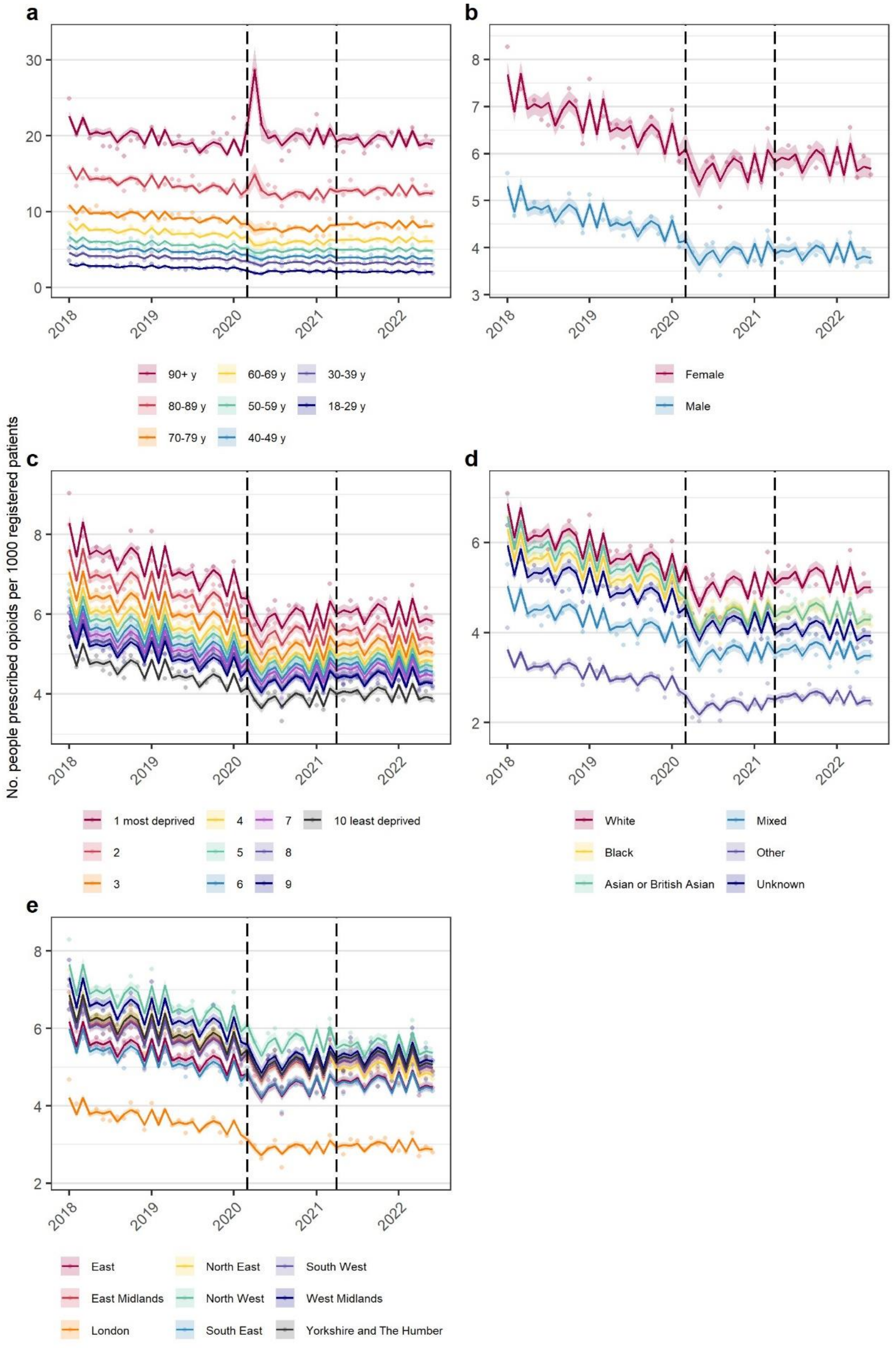

Supplementary Table 1. Changes in number of people prescribed opioids during lockdown and recovery periods by demographic categories

	Pre-COVID-19 median monthly prevalence per 1000, IQR	Lockdown period (Mar 2020-Mar 2021)		Recovery period (Apr 2021-Jun 2022)	
		Median monthly prevalence per 1000, IQR	Level shift (%, 95% CI)	Median monthly prevalence per 1000, IQR	Level shift (%, 95% CI)
**Full cohort**	50.9 (49.6-51.7)	49.1 (47.6-49.7)	--	48.2 (47.2-48.9)	--
**Age**					
18-29 y	8.1 (7.9-8.6)	7.5 (7.0-7.6)	-5.2 (-8.8, -1.4)	7.0 (6.8-7.2)	-4.2 (-7.2, -1.2)
30-39 y	19.9 (19.3-20.4)	19.0 (18.4-19.3)	0.0 (-3.4, 3.6)	18.6 (18.4-19.0)	0.0 (-3.0, 3.1)
40-49 y	37.3 (36.5-38.2)	35.8 (34.4-36.0)	-0.1 (-3.4, 3.2)	34.5 (34.0-35.2)	-1.4 (-3.9, 1.2)
50-59 y	56.5 (55.0-57.1)	55.3 (52.9-55.7)	2.6 (-0.8, 6.1)	54.7 (54.0-55.8)	1.5 (-1.4, 4.6)
60-69 y	81.3 (79.2-82.8)	77.9 (75.3-79.2)	0.2 (-3.1, 3.6)	77.0 (75.5-77.9)	0.9 (-1.9, 3.8)
70-79 y	100.7 (98.1-103.1)	95.2 (92.2-97.1)	-1.3 (-4.8, 2.3)	93.9 (91.9-95.0)	0.7 (-2.3, 3.8)
80-89 y	130.6 (126.8-132.3)	125.6 (121.9-127.3)	0.2 (-3.4, 3.9)	122.8 (119.5-123.5)	0.1 (-2.8, 3.1)
90+ y	146.7 (143.7-148.8)	144.7 (142.4-145.7)	2.4 (-1.3, 6.3)	140.5 (137.8-142.7)	0.0 (-2.4, 2.5)
**Sex**					
Female	62.6 (61.1-63.6)	60.8 (58.8-61.3)	0.0 (-3.3, 3.4)	59.7 (58.8-60.8)	0.1 (-2.8, 3.0)
Male	38.9 (37.9-39.7)	37.2 (36.2-37.9)	-1.1 (-4.4, 2.4)	36.3 (35.5-36.8)	-1.3 (-4.0, 1.5)
**IMD decile**					
1 most deprived	78.7 (77.1-80.5)	75.5 (73.1-76.5)	-0.7 (-3.7, 2.4)	73.8 (72.7-74.9)	-0.4 (-3.0, 2.2)
2	67.1 (65.5-68.2)	64.7 (62.6-65.6)	0.0 (-3.1, 3.2)	63.6 (62.5-64.4)	0.0 (-2.6, 2.6)
3	59.8 (58.6-60.9)	57.8 (55.9-58.3)	0.1 (-3.0, 3.3)	56.6 (55.5-57.3)	-0.4 (-2.9, 2.3)
4	53.2 (51.9-54.1)	51.5 (49.9-52.1)	0.7 (-2.5, 3.9)	50.6 (49.6-51.4)	0.0 (-2.7, 2.6)
5	50.0 (49.0-50.9)	48.7 (47.4-49.1)	1.0 (-2.2, 4.3)	47.7 (47.0-48.6)	0.0 (-2.7, 2.7)
6	45.9 (44.9-46.7)	44.9 (43.5-45.4)	1.4 (-1.8, 4.7)	44.1 (43.4-44.8)	0.2 (-2.5, 3.0)
7	43.4 (42.5-44.2)	42.4 (41.1-42.8)	1.2 (-2.0, 4.6)	41.7 (40.8-42.3)	0.1 (-2.6, 2.9)
8	41.1 (40.0-41.8)	39.7 (38.5-40.1)	0.5 (-2.6, 3.7)	39.1 (38.3-39.8)	0.2 (-2.5, 3.0)
9	38.1 (37.3-38.7)	37.3 (36.1-37.6)	1.4 (-1.9, 4.9)	36.5 (36.0-37.4)	0.2 (-2.5, 3.0)
10 least deprived	31.6 (30.8-32.2)	30.5 (29.4-30.9)	0.2 (-2.9, 3.5)	30.1 (29.6-30.6)	0.8 (-2.1, 3.7)
**Ethnicity**					
White	55.6 (54.3-56.2)	54.4 (52.6-54.9)	2.6 (-1.0, 6.4)	53.8 (53.1-54.8)	1.4 (-1.6, 4.4)
Asian or British Asian	28.7 (27.5-29.3)	26.0 (24.5-26.3)	-5.7 (-8.5, -2.8)	24.8 (24.6-25.4)	-1.7 (-4.7, 1.4)
Black	28.1 (27.0-29.0)	26.1 (25.2-27.1)	-2.5 (-5.5, 0.6)	25.7 (24.7-26.1)	-0.8 (-3.7, 2.2)
Mixed	25.0 (24.1-25.6)	23.7 (22.6-24.3)	-1.1 (-4.3, 2.1)	23.3 (22.9-23.9)	0.5 (-2.2, 3.3)
Other	19 .0(18.1-19.7)	17.6 (16.6-17.7)	-4.2 (-7.2, -1.2)	17.0 (16.7-17.3)	-0.7 (-3.5, 2.2)

Unknown	46.7 (45.2-48.5)	43.0 (41.6-43.6)	-3.8 (-7.4, 0.0)	41.0 (40.2-41.5)	-3.5 (-6.2, -0.8)
**Region**					
East	45.4 (44.3-46.2)	43.9 (42.7-44.4)	-0.6 (-3.7, 2.7)	43.1 (42.6-44.0)	-0.1 (-2.7, 2.6)
East Midlands	55.1 (53.8-56.0)	54.0 (52.4-54.6)	0.9 (-2.3, 4.2)	53.4 (52.5-54.3)	0.2 (-2.4, 2.9)
London	20.7 (20.0-21.4)	18.9 (18.2-19.4)	-5.8 (-8.9, -2.5)	18.3 (17.9-18.7)	-2.4 (-5.0, 0.4)
North East	68.4 (67.1-69.8)	66.3 (64.2-67.1)	-0.6 (-3.6, 2.5)	65.0 (63.5-65.4)	-1.1 (-3.6, 1.4)
North West	70.2 (68.6-72.0)	67.0 (65.3-68.1)	-1.7 (-4.9, 1.7)	64.7 (63.4-65.5)	-3.0 (-5.4, -0.6)
South East	42.2 (41.2-42.9)	41.1 (39.6-41.6)	0.2 (-3.1, 3.5)	40.1 (39.4-41.1)	-0.7 (-3.4, 2.0)
South West	47.0 (45.9-47.8)	45.9 (44.3-46.2)	0.3 (-2.9, 3.5)	44.7 (43.9-45.6)	-0.7 (-3.3, 2.0)
West Midlands	56.0 (54.6-56.9)	54.8 (52.4-55.3)	0.6 (-2.6, 3.8)	54.8 (54.3-56.0)	1.8 (-1.3, 4.9)
Yorkshire and The Humber	57.0 (55.8-58.0)	55.1 (53.3-55.6)	-0.7 (-3.6, 2.4)	54.4 (53.4-55.1)	-0.1 (-2.7, 2.7)

Supplementary Table 2. Changes in number of people newly prescribed opioids during lockdown and recovery periods by demographic categories

		Lockdown period (Mar 2020-Mar 2021)		Recovery period (Apr 2021-Jun 2022)	
	**Pre-COVID-19 median monthly prevalence per 1000, IQR**	**Median monthly prevalence per 1000, IQR**	**Level shift (95% CI)**	**Median monthly prevalence per 1000, IQR**	**Level shift (95% CI)**
**Full cohort**	5.7 (5.4-5.9)	4.9 (4.6-4.9)		4.8 (4.6-5.0)	
**Age**					
18-29 y	2.7 (2.5-2.9)	2.2 (2.0-2.2)	-13.0 (-20.2, -5.2)	2.1 (2.0-2.1)	-1.0 (-7.8, 6.4)
30-39 y	4.0 (3.8-4.1)	3.4 (3.1-3.4)	-9.6 (-16.8, -1.8)	3.1 (3.1-3.3)	0.7 (-6.4, 8.4)
40-49 y	4.8 (4.6-5.1)	4.1 (4.0-4.2)	-8.8 (-16.4, -0.5)	3.9 (3.7-4.0)	0.3 (-6.6, 7.7)
50-59 y	5.7 (5.5-6.0)	4.9 (4.7-5.1)	-7.0 (-14.5, 1.1)	4.9 (4.8-5.1)	4.4 (-2.4, 11.8)
60-69 y	7.4 (7.0-7.5)	5.9 (5.6-6.3)	-10.9 (-18.5, -2.6)	6.2 (6.0-6.4)	8.4 (0.6, 16.9)
70-79 y	9.5 (9.0-9.9)	7.7 (7.5-8.3)	-10.4 (-18.9, -1.1)	8.2 (7.9-8.5)	10.8 (1.6, 21.0)
80-89 y	13.7 (13.0-14.4)	12.3 (12.0-13.2)	-2.6 (-12.0, 7.9)	12.5 (12.2-12.8)	7.2 (-0.5, 15.4)
90+ y	19.5 (18.5-20.3)	20.2 (19.9-21.4)	10.5 (-3.0, 25.9)	19.4 (18.8-20.0)	1.5 (-5.9, 9.6)
**Sex**					
Male	6.7 (6.4-7.0)	5.8 (5.6-5.9)	-7.7 (-15.9, 1.4)	5.8 (5.6-6.0)	5.7 (-1.6, 13.7)
Female	4.7 (4.5-4.8)	3.9 (3.7-4.1)	-9.0 (-17.3, 0.2)	3.9 (3.7-4.0)	3.5 (-3.6, 11.1)
**IMD decile**					
1 most deprived	7.2 (7.0-7.5)	6.1 (5.6-6.2)	-10.1 (-17.0, -2.7)	5.9 (5.8-6.3)	4.6 (-2.4, 12.1)
2	6.7 (6.4-6.9)	5.7 (5.3-5.7)	-9.5 (-16.8, -1.6)	5.5 (5.3-5.7)	4.2 (-2.6, 11.5)
3	6.2 (5.9-6.4)	5.3 (4.9-5.3)	-9.2 (-16.5, -1.2)	5.1 (5.0-5.4)	5.0 (-2.0, 12.5)
4	5.8 (5.5-6.1)	4.9 (4.7-5.0)	-8.2 (-15.7, -0.1)	4.9 (4.8-5.1)	5.4 (-1.5, 12.7)
5	5.7 (5.4-5.8)	4.8 (4.6-5.0)	-6.9 (-14.5, 1.4)	4.8 (4.6-5.0)	4.7 (-2.0, 11.9)
6	5.4 (5.1-5.5)	4.7 (4.5-4.8)	-6.7 (-14.7, 1.9)	4.7 (4.6-4.8)	5.5 (-1.2, 12.7)
7	5.3 (5.0-5.4)	4.6 (4.4-4.7)	-6.1 (-14.1, 2.6)	4.6 (4.4-4.7)	5.4 (-1.3, 12.7)
8	5.1 (4.9-5.3)	4.3 (4.2-4.5)	-7.1 (-14.9, 1.4)	4.4 (4.3-4.5)	5.6 (-0.9, 12.6)
9	5.0 (4.8-5.2)	4.3 (4.2-4.5)	-6.0 (-14.1, 2.9)	4.3 (4.2-4.5)	5.5 (-0.9, 12.4)
10 least deprived	4.6 (4.4-4.8)	3.9 (3.7-4.0)	-7.2 (-15.1, 1.5)	3.9 (3.9-4.1)	6.8 (0.2, 13.9)
**Ethnicity**					
White	5.9 (5.6-6.1)	5.1 (4.9-5.2)	-6.5 (-14.6, 2.4)	5.1 (5.0-5.3)	6.0 (-0.4, 12.9)
Asian or British Asian	5.7 (5.4-5.8)	4.5 (4.2-4.6)	-15.1 (-21.4, -8.3)	4.4 (4.2-4.7)	4.4 (-3.4, 12.9)
Black	5.4 (5.1-5.7)	4.5 (4.3-4.7)	-10.5 (-18.3, -1.8)	4.5 (4.2-4.6)	3.2 (-3.2, 10.1)
Mixed	4.3 (4.1-4.5)	3.6 (3.4-3.8)	-9.6 (-17.5, -1.0)	3.6 (3.5-3.8)	4.3 (-1.9, 10.9)
Other	3.1 (3.0-3.2)	2.4 (2.4-2.5)	-15.7 (-22.3, -8.5)	2.5 (2.5-2.6)	10.5 (2.1, 19.7)
Unknown	5.1 (4.8-5.3)	4.3 (4.0-4.4)	-9.9 (-18.3, -0.6)	4.0 (3.9-4.2)	-0.3 (-6.0, 5.8)

**Region**					
East	5.4 (5.1-5.5)	4.6 (4.4-4.7)	-9.0 (-16.7, -0.7)	4.6 (4.4-4.7)	5.8 (-0.8, 12.9)
East Midlands	5.9 (5.7-6.1)	5.1 (4.8-5.2)	-7.2 (-14.8, 1.1)	5.1 (4.9-5.2)	5.7 (-1.1, 12.9)
London	3.7 (3.5-3.9)	2.9 (2.9-3.0)	-13.3 (-20.7, -5.1)	2.9 (2.8-3.1)	4.5 (-2.3, 11.9)
North East	6.0 (5.8-6.2)	5.1 (4.7-5.2)	-8.9 (-16.5, -0.6)	4.9 (4.5-5.3)	1.6 (-4.8, 8.5)
North West	6.7 (6.4-6.9)	5.7 (5.4-5.9)	-7.4 (-15.2, 1.1)	4.9 (4.7-5.1)	0.5 (-5.3, 6.7)
South East	5.2 (5.0-5.4)	4.5 (4.3-4.7)	-5.7 (-14.0, 3.5)	5.4 (5.3-5.7)	4.2 (-2.2, 11.1)
South West	5.8 (5.6-6.0)	5.1 (4.9-5.2)	-5.8 (-13.7, 2.7)	4.5 (4.4-4.7)	5.1 (-1.5, 12.2)
West Midlands	6.4 (6.1-6.6)	5.3 (4.9-5.4)	-10.8 (-17.8, -3.2)	5.0 (5.0-5.3)	4.8 (-2.4, 12.6)
Yorkshire and the Humber	6.0 (5.8-6.2)	5.2 (4.8-5.3)	-7.4 (-14.6, 0.5)	5.2 (5.1-5.6)	5.7 (-1.4, 13.2)

Supplementary Figure 5. All-cause mortality by age group and care home status, from Schultze et al. Mortality among Care Home Residents in England during the first and second waves of the COVID-19 pandemic: an observational study of 4.3 million adults over the age of 65. *Lancet Reg Health Eur*. 2022 Mar; 14: 100295. doi: 10.1016/j.lanepe.2021.100295

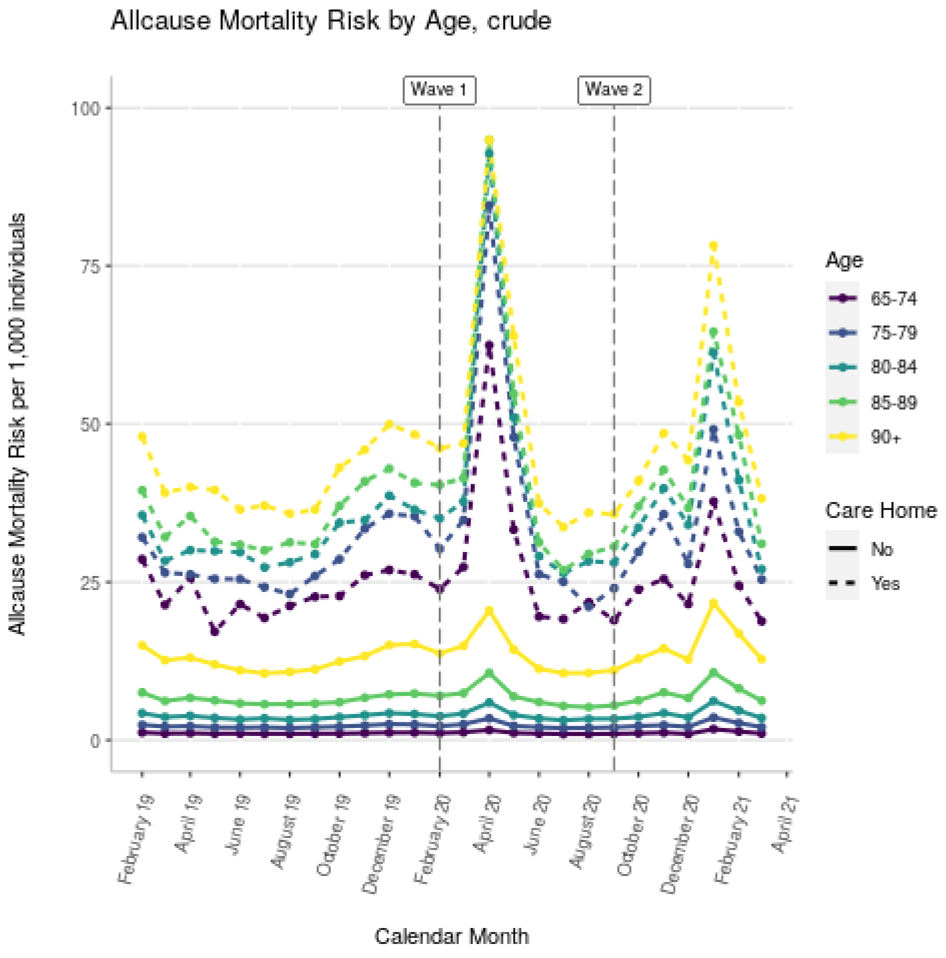

Information governance

NHS England is the data controller of the NHS England OpenSAFELY COVID-19 Service; TPP is the data processor; all study authors using OpenSAFELY have the approval of NHS England.(1) This implementation of OpenSAFELY is hosted within the TPP environment which is accredited to the ISO 27001 information security standard and is NHS IG Toolkit compliant.(2)

Patient data has been pseudonymised for analysis and linkage using industry standard cryptographic hashing techniques; all pseudonymised datasets transmitted for linkage onto OpenSAFELY are encrypted; access to the NHS England OpenSAFELY COVID-19 service is via a virtual private network (VPN) connection; the researchers hold contracts with NHS England and only access the platform to initiate database queries and statistical models; all database activity is logged; only aggregate statistical outputs leave the platform environment following best practice for anonymisation of results such as statistical disclosure control for low cell counts.(3)

The service adheres to the obligations of the UK General Data Protection Regulation (UK GDPR) and the Data Protection Act 2018. The service previously operated under notices initially issued in February 2020 by the Secretary of State under Regulation 3(4) of the Health Service (Control of Patient Information) Regulations 2002 (COPI Regulations), which required organisations to process confidential patient information for COVID-19 purposes; this set aside the requirement for patient consent.(4) As of 1 July 2023, the Secretary of State has requested that NHS England continue to operate the Service under the COVID-19 Directions 2020.(5) In some cases of data sharing, the common law duty of confidence is met using, for example, patient consent or support from the Health Research Authority Confidentiality Advisory Group.(6)

Taken together, these provide the legal bases to link patient datasets using the service. GP practices, which provide access to the primary care data, are required to share relevant health information to support the public health response to the pandemic, and have been informed of how the service operates.

(1) NHS Digital. The NHS England OpenSAFELY COVID-19 service - privacy notice [Internet]. 2023 [cited 2023 Jul 5]. Available from: https://digital.nhs.uk/coronavirus/coronavirus-covid-19-response-information-governance-hub/the-nhs-england-opensafely-covid-19-service-privacy-notice

(2) NHS Digital. NHS Digital. 2023 [cited 2023 Jul 5]. Data Security and Protection Toolkit. Available from: https://digital.nhs.uk/data-and-information/looking-after-information/data-security-and-information-governance/data-security-and-protection-toolkit

(3) NHS Digital [Internet]. [cited 2023 Mar 6]. ISB1523: Anonymisation Standard for Publishing Health and Social Care Data. Available from: https://digital.nhs.uk/data-and-information/information-standards/information-standards-and-data-collections-including-extractions/publications-and-notifications/standards-and-collections/isb1523-anonymisation-standard-for-publishing-health-and-social-care-data

(4) UK Department of Health and Social Care. GOV.UK. 2022 [cited 2023 Jul 5]. [Withdrawn] Coronavirus (COVID-19): notice under regulation 3(4) of the Health Service (Control of Patient Information) Regulations 2002 – general. Available from: https://www.gov.uk/government/publications/coronavirus-covid-19-notification-of-data-controllers-to-share-information/coronavirus-covid-19-notice-under-regulation-34-of-the-health-service-control-of-patient-information-regulations-2002-general--2

(5) NHS Digital. NHS Digital. 2022 [cited 2023 Jul 5]. Secretary of State for Health and Social Care: COVID-19 Public Health Directions 2020. Available from: https://digital.nhs.uk/about-nhs-digital/corporate-information-and-documents/directions-and-data-provision-notices/secretary-of-state-directions/covid-19-public-health-directions-2020

(6) NHS Health Research Authority. Health Research Authority. [cited 2023 Jul 5]. Confidentiality Advisory Group. Available from: https://www.hra.nhs.uk/about-us/committees-and-services/confidentiality-advisory-group/

Patient and public involvement

We have involved patients and the public in various ways: we developed a public website that provides a detailed description of the platform in language suitable for a lay audience (https://opensafely.org); we have participated in two citizen juries exploring public trust in OpenSAFELY; we co-developed an explainer video; we have patient representation who are experts by experience on our OpenSAFELY Oversight Board; we have partnered with Understanding Patient Data to produce lay explainers on the importance of large datasets for research; we have presented at various online public engagement events to key communities; and more. To ensure the patient voice is represented, we are working closely to decide on language choices with appropriate medical research charities.

Software and reproducibility

Data management was performed using Python 3.8, with analysis carried out using R 4.0.5. Code for data management and analysis as well as codelists archived online (https://github.com/opensafely/opioids-covid-research).
